# Characterization of Bimi^®^ Broccoli as a Convenience Food: Nutritional Composition and Quality Traits Following Industrial Sous-Vide Processing

**DOI:** 10.3390/molecules30153255

**Published:** 2025-08-03

**Authors:** Elisa Canazza, Christine Mayr Marangon, Dasha Mihaylova, Valerio Giaccone, Anna Lante

**Affiliations:** 1 Department of Agronomy, Food, Natural Resources, Animals, and Environment—DAFNAE, University of Padova, Viale dell’Università, 16, 35020 Legnaro, Italy; elisa.canazza.3@phd.unipd.it (E.C.); christine.marangon@unipd.it (C.M.M.); 2 Department of Microbiology and Biotechnology, University of Food Technologies, 26 Maritza Blvd., 4002 Plovdiv, Bulgaria; dashamihaylova@yahoo.com; 3 Department of Animal Medicine, Productions and Health, University of Padova, Viale dell’Università, 16, 35020 Legnaro, Italy; valerio.giaccone@unipd.it

**Keywords:** *Brassica oleracea* Italica × Alboglabra, fifth-range vegetables, convenience food, nutritional compounds, antioxidant capacity, shelf life, sensory profile

## Abstract

This study investigates Bimi^®^ (*Brassica oleracea* Italica × Alboglabra), a hybrid between kailan and conventional broccoli, to evaluate its compositional, functional, and sensory properties in relation to industrial sous-vide processing and refrigerated storage. Proximate composition, amino acid and fatty acid profiles, and mineral content were determined in raw samples. Color, chlorophyll content, total polyphenols, and antioxidant capacity (FRAP, ABTS, DPPH) were analyzed before and after sous-vide treatment and following 60 days of storage. Microbiological and physicochemical stability was monitored over 90 days under standard (4 °C) and mildly abusive (6–10 °C) storage conditions. Sensory profiling of Bimi^®^ and conventional broccoli was performed on sous-vide samples. The results showed an increase in total polyphenols and antioxidant activity after processing, while chlorophylls decreased. Microbiological safety was maintained under all conditions, with stable water activity and only moderate acidification. Bimi^®^ provided a valuable source of protein (4.32 g/100 g FW, 8.63% RDA), appreciable amounts of dietary fiber (2.96 g/100 g FW, 11.85% RDA), and essential minerals such as potassium (15.59% RDA), phosphorus (14.05% RDA), and calcium (8.09% RDA). Sensory evaluation revealed a milder flavor profile than that of conventional broccoli, accompanied by an asparagus-like aroma. These findings support the suitability of Bimi^®^ for industrial sous-vide processing and its potential as a nutritious convenience food.

## 1. Introduction

The *Brassica* genus, belonging to the Brassicaceae (or Cruciferae) family, comprises numerous cultivated species of notable agricultural importance, among which *Brassica oleracea* is particularly prominent. This species includes several well-known vegetables such as broccoli, cabbage, cauliflower, kohlrabi, and mustard. These crops are valued as rich sources of health-promoting bioactive compounds, including potentially protective phytochemicals such as vitamins (C, A, E, etc.), minerals, carotenoids, chlorophylls, phenolic compounds, folates, glucosinolates and their breakdown products, isothiocyanates, and indoles [1,2,3,4,5,6]. Broccoli has been among the main *Brassica* species studied for their bioactive compounds and their potential health benefits, particularly focusing on glucosinolates and their derived products [7,8]. Despite its excellent nutritional profile and the growing consumer interest in its health-promoting properties, broccoli often faces limited acceptance due to its characteristic sulfurous and pungent aroma and bitter taste. This has led to the selection of broccoli varieties with a less intense flavor than conventional ones to increase their consumption [9,10]. Bimi^®^ is a natural hybrid between kailan (*Brassica oleracea*, Alboglabra group) and conventional broccoli (*B. oleracea*, Italica group) [11,12], initially developed by Sakata Seed Company (Japan) for its improved sensory attributes, including a milder aroma, and a taste that is slightly sweeter than conventional broccoli [9]. Initially marketed in Mexico in 1994 as Asparation^®^, the hybrid was later introduced to the U.S. and gradually expanded to markets in Brazil, Australia, and northern Europe. In Europe, Spain leads production from autumn to spring, while cultivation shifts to the UK and Netherlands in summer [10]. Bimi^®^ has been available in Italy since 2017 under exclusive license by Cinana Vibes, with local production starting in 2022. Bimi^®^ morphologically resembles a small broccoli, but is distinguished by its smaller florets, and long and thin stem with a sweet taste, like that of green asparagus [10,13]. The stem is also tender and completely edible [2,14].

The fast-paced nature of contemporary life has significantly curtailed the time available for meal preparation, thereby fueling the demand for foods that are both convenient and nutritionally balanced. Consumers are increasingly seeking practical solutions that align with health and wellness goals, favoring ready-to-eat and ready-to-cook products [15,16,17,18]. These foods are expected to offer a favorable nutritional profile, be free from synthetic additives, and contain bioactive compounds with high antioxidant potential—characteristics underpinning their growing popularity across both the retail and foodservice sectors [9,19]. While certain cruciferous vegetables are suitable for raw consumption, most *Brassica* species typically require cooking, which induces changes in their nutritional composition, sensory properties, and physicochemical characteristics [2,20]. Sous-vide cooking, introduced by French chef Georges Pralus in the mid-1970s, involves vacuum-sealing food inside hermetically sealed heat-stable vacuum bags and cooking it under controlled temperature (65–95 °C) and time (1–7 h) conditions [19]. Meat and fish products are generally processed below 70 °C, while vegetables are processed above 85 °C [21]. This method ensures efficient heat transfer from water to food, minimizing moisture and nutrient loss while preserving volatile compounds. It also limits oxidative changes and inhibits the growth of aerobic bacteria, enhancing food safety and extending shelf life during refrigerated storage [19,22]. Products not for immediate consumption rely on pasteurizing heat treatment followed by chilled storage for safety and preservation [19,23]. Sous-vide cooking—characterized by vacuum packaging, precisely controlled heat treatment, and rapid cooling—requires the selection of appropriate microbiological targets based on the food type and intended refrigerated shelf life [19]. For products with a shelf life of ≤10 days, a heat treatment equivalent to 70 °C for 2 min at the core is recommended to achieve a 6–log reduction in *Listeria monocytogenes*. For products stored for more than 10 days, a more intensive treatment of 90 °C for 10 min at the core is advised to inactivate spores of *Clostridium botulinum* Group II [24,25,26,27].

Although previous studies have investigated the postharvest quality of Bimi^®^ broccoli [9,28,29], little is known about its behavior under industrial sous-vide processing and subsequent refrigerated storage. To the best of our knowledge, no previous research has directly compared the sensory profile of Bimi^®^ and conventional broccoli subjected to the same thermal treatment. This study addresses this gap by evaluating not only the compositional and sensory properties, but also the potential of Bimi^®^ broccoli as a convenience food with extended shelf life. The findings offer valuable insights into its suitability for developing health-oriented foods that meet modern consumer expectations for quality, functionality, and convenience.

Therefore, the present study aimed to (i) characterize the proximate composition and the mineral, amino acid, and fatty acid profiles of raw Bimi^®^; (ii) evaluate the effects of industrial sous-vide processing and refrigerated storage on color, chlorophyll content, total phenolics, and antioxidant capacity (FRAP, ABTS, DPPH); (iii) assess the microbiological and physicochemical stability of sous-vide Bimi^®^ over 90 days under standard and abusive temperature conditions; (iv) compare the sensory attributes of sous-vide Bimi^®^ with those of conventional broccoli processed under the same industrial conditions. The comprehensive profiling of bioactive compounds of nutritional and functional interest—particularly glucosinolates and their hydrolysis products—represents a direction for future research aimed at fully exploring the biofunctional potential of such products.

## 2. Results and Discussion

### 2.1. Compositional Characterization of Raw Bimi^®^ Broccoli

#### 2.1.1. Proximate Composition

The proximate composition of raw Bimi^®^ broccoli is presented in Table 1. Parameters were consistent with data previously reported by Rosés et al. (2023) [14], except for slightly lower protein and carbohydrate contents, which may be attributed mainly to agronomic or environmental variables. The table also reports the Recommended Dietary Allowance (RDA) according to European legislation [30] and EFSA recommendations [31].

As expected for vegetable products, Bimi^®^ had a high moisture of 89.39 ± 0.56%, a level typical of other *Brassica oleracea* varieties. Similar values have been reported in *B. oleracea* var. *capitata* (87.93%), var. *acephala* (81.38%), var. *botrytis* (90.62%), and var. *italica* (87.25%) (Rizwan & Masoodi, 2025) [32]. The protein content was 4.32 ± 0.06 g/100 g FW, which is relatively high compared to other cruciferous vegetables such as broccoli (2.57 g/100 g FW), cabbage (1.28 g/100 g FW), cauliflower (1.92 g/100 g FW), Brussels sprouts (3.38 g/100 g FW), kale (2.92 g/100 g FW), and collards (3.02 g/100 g FW) [33]. According to European legislation [34], food is considered a source of protein when at least 12% of its energy value is derived from protein. In Bimi^®^, proteins contribute approximately 48.7% of the total energy. Dietary fiber was present at 2.96 ± 0.03 g/100 g FW in line with values reported for other vegetables in the Brassicaceae family [33]. According to EFSA guidelines, the recommended daily intake of dietary fiber is at least 25 g [31]; a single 150 g portion of Bimi^®^ would provide approximately 18% of this amount. The negligible fat content (0.46 ± 0.04%) and low carbohydrate content (2.03 ± 0.12%) in Bimi^®^ are particularly suitable for individuals following low-fat diet regimens [32,33]. The ash content was 0.84 ± 0.01%, reflecting the typical level of inorganic components found in vegetables [35]. The overall energy value was low, measured at 35.46 ± 0.14 kcal/100 g FW [34], which is consistent with the energy density typically reported for cruciferous vegetables, ranging from 11 to 48 kcal/100 g, due to their high water content [33].

#### 2.1.2. Mineral Content

The mineral content of Bimi^®^ broccoli was determined for six essential macro minerals—calcium (Ca), potassium (K), magnesium (Mg), sodium (Na), phosphorus (P), and sulfur (S)—and is expressed as mg per 100 g of FW (Table 2). Analyses were conducted separately on the whole edible portion, as well as on isolated florets and stems.

As also reported by Martínez–Hernández et al. (2015) [36] in kailan-hybrid broccoli after harvest, the contents of calcium, magnesium, and phosphorus were higher in florets compared to stems. In contrast, potassium and sodium were more abundant in the stem fraction. In the present study, sulfur content was also significantly higher in the florets. A 100 g portion of whole Bimi^®^ broccoli provides appreciable amounts of essential macro minerals: potassium (15.59% RDA), phosphorus (14.05% RDA), and calcium (8.09% RDA). Magnesium also contributes modestly (4.95% RDA), while sodium accounts for less than 1% (0.55% RDA) [30]. Compared to the sodium concentrations (mg/100 g FW) reported by Xiao et al. (2016) [37] for 30 commercially grown microgreens of the Brassicaceae family, ranging from 19 to 68 mg/100 g FW, the Bimi^®^ samples analyzed in this study showed lower sodium levels. The variations observed compared to the literature may result from a range of factors, including genotype-specific traits, agronomic practices, environmental and soil conditions, plant maturity at harvest, and differences in analytical methodologies [38,39,40].

#### 2.1.3. Amino Acid Profile

The amino acid profile of raw Bimi^®^ broccoli, expressed as mg/100 g FW, is reported in Table 3.

Given the limited information available on the amino acid composition of Bimi^®^ broccoli, particularly regarding its anatomical differentiation, the present study quantified eighteen essential (EAA) and non-essential (NEAA) amino acids [41,42] in the whole edible portion as well as in isolated florets and stems, to provide a more comprehensive nutritional profile. Statistically significant differences in total free amino acid content were observed among the anatomical fractions, with higher levels detected in florets (4470.92 ± 196.52 mg/100 g FW) compared to stems (3353.83 ± 193.11 mg/100 g FW). This distributional trend was consistent across both essential (EAAs) and non-essential amino acids (NEAAs). The whole sample showed intermediate values, as expected given that it represents the intact edible portion prior to separation, comprising both floral and stem tissues, and thus reflects the overall amino acid profile of the Bimi^®^ broccoli. Florets exhibited higher concentrations of all essential amino acids compared to stems, with particularly elevated levels of lysine (276.87 ± 20.23 mg/100 g FW), leucine (203.50 ± 1.48 mg/100 g FW), and threonine (140.43 ± 12.56 mg/100 g FW). Florets exhibited higher levels of all essential amino acids relative to stems. These findings are consistent with previous observations in *Brassica oleracea* var. *italica*, such as those reported by Liu et al. (2018) [43], which similarly documented higher amino acid concentrations in inflorescence tissues relative to vegetative parts. Beyond total abundance, all tissues were dominated by glutamic and aspartic acids, which together accounted for the largest proportion of the free amino acid pool. Glutamic acid was the most abundant across all fractions, ranging from 1618.68 mg/100 g FW in florets to 1743.10 mg/100 g FW in stems, followed by aspartic acid, which ranged from 405.78 to 520.39 mg/100 g FW. This compositional trend is consistent with previous reports in *Brassica oleracea* cultivars [41,44,45,46], where glutamic and aspartic acids are consistently identified as the predominant constituents of the free amino acid profile.

#### 2.1.4. Fatty Acid Profile

**Figure 1:**
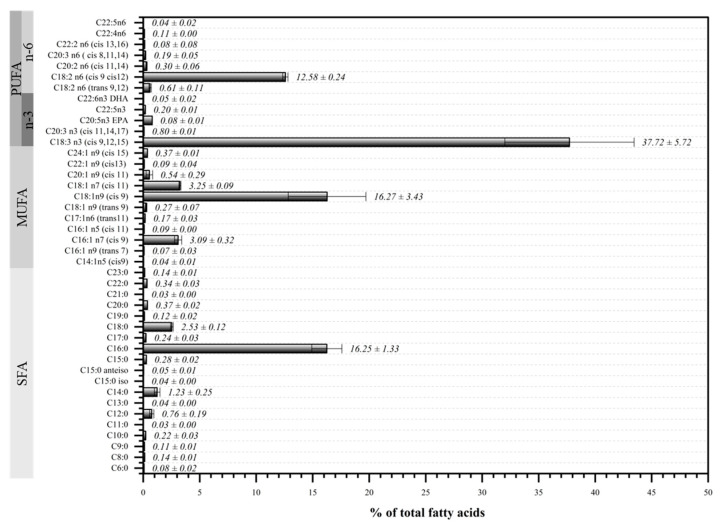
Fatty acid profile of Bimi^®^ broccoli (% of total fatty acids). Data are mean ± SD (*n* = 3). Fatty acids are grouped as saturated (SFAs), monounsaturated (MUFAs), and polyunsaturated (PUFAs), with omega-3 and omega-6 fractions indicated.

The fatty acid profile of Bimi^®^ broccoli is illustrated in Figure 1.

The results show a clear predominance of unsaturated fatty acids (77.02 ± 1.71%) over saturated ones (22.98 ± 1.71%). Within the unsaturated fraction, polyunsaturated fatty acids (PUFAs) dominate (52.76 ± 5.97%), with omega-3 and omega-6 fatty acids contributing 38.85 ± 5.73% and 13.91 ± 0.24%, respectively. Palmitic acid (C16:0) is the most abundant saturated fatty acid, accounting for 70.68 ± 0.53% of the saturated fatty acid (SFA) fraction. Among the unsaturated fatty acids, alpha-linolenic acid (C18:3 n3, cis 9,12,15) is predominant (48.91 ± 6.34%), followed by oleic acid (C18:1 n9, cis 9) at 21.18 ± 4.93%.

These findings are consistent with Bhandari & Kwak (2015) [47], who reported palmitic (23.52–38.42%), linoleic (13.09–18.97%), and linolenic acids (26.32–51.80%) as the predominant fatty acids in *Brassica oleracea* L. var. *acephala* across several cauliflower and broccoli cultivars.

### 2.2. Color Traits and Chlorophyll Content

The impact of sous-vide processing and subsequent refrigerated storage on the color traits and chlorophyll content of Bimi^®^ broccoli was assessed through instrumental color measurements and spectrophotometric pigment quantification. As shown in Table 4, significant differences in color parameters (L*, a*, b*, C*, *H*°) were observed between the stem and floret tissues in the raw samples.

**Figure 2:**
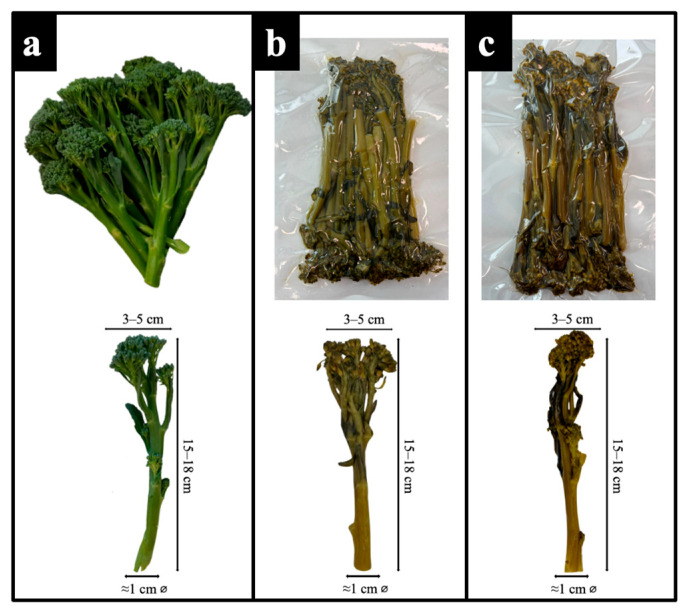
Visual appearance of Bimi^®^ broccoli at three stages: (**a**) raw, (**b**) after one day (t_1_), and (**c**) after 60 days (t_60_) of refrigerated storage at 4 °C post sous-vide processing.

Stems exhibited higher lightness (L* = 48.56 ± 0.38), stronger green hue (a* = −20.06 ± 0.38), and more intense yellow chroma (b* = 33.27 ± 0.11) compared to florets (L* = 42.75 ± 0.38; a* = −12.65 ± 0.51; b* = 20.28 ± 0.32). Consequently, chroma (C*) and hue angle (*H°*) were also greater in stems (C* = 38.85 ± 0.15; *H°* = 120.68 ± 0.44), indicating a more vivid and bright appearance. These findings are consistent with Martínez–Hernández et al. (2013) [2,7], who reported a higher degree of greenness and brightness in stems compared to florets in kailan-hybrid broccoli. After sous-vide processing, a marked reduction in brightness and greenness was evident in both tissues. At t_1_, L* values dropped significantly (stems: 40.30 ± 0.23; florets: 37.35 ± 0.28), while a* values increased (less negative), indicating a loss of greenness. This trend was accompanied by a rise in b* values, suggesting increased yellowness. After 60 days of refrigerated storage (t_60_), these shifts became even more pronounced. *H°* and lightness continued to decrease, reflecting an overall darkening of the tissue (Figure 2), while total color difference (Δ*E*) increased significantly, surpassing the perceptibility threshold of 3.0 [48] after sous-vide processing and reaching Δ*E* = 17.54 ± 0.38 in stems and 16.73 ± 0.32 in florets at t_60_.

These visual and instrumental observations are directly linked to the degradation of chlorophyll [49,50], which was evaluated. Chlorophyll losses are primarily attributed to cellular breakdown, pigment degradation, and the formation of pheophytins [51]. The extent of chlorophyll degradation largely depends on the temperature of thermal processing and the presence of organic acids released during cooking, which can accelerate pigment breakdown [49]. According to the literature, chlorophyll loss brought about by thermal processing varies from 12 to 66%, depending on the species, the edible part of the plant, and the time and temperature of the treatment [52,53].

**Figure 3:**
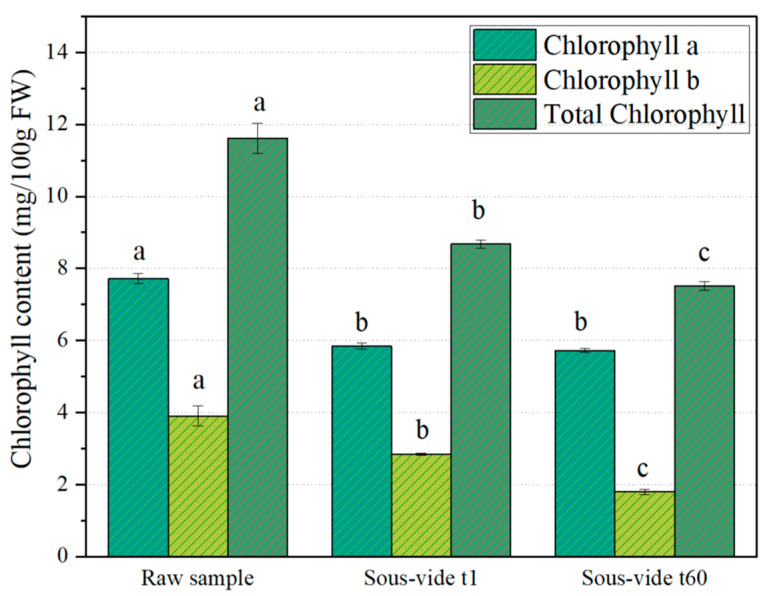
Chlorophyll content (mg/100 g FW) in Bimi^®^ broccoli: raw, after one day (t_1_), and after 60 days (t_60_) of refrigerated storage at 4 °C post sous-vide processing. Values are mean ± SD (*n* = 3). Different letters indicate significant differences (*p* ≤ 0.05, ANOVA + Tukey’s test).

As reported in Figure 3, the raw samples contained a total chlorophyll content of 11.62 ± 0.42 mg/100 g FW, with chlorophyll a being the dominant form (7.72 ± 0.14 mg/100 g FW) compared to chlorophyll b (3.90 ± 0.28 mg/100 g FW). The sous-vide processing caused a significant loss of total chlorophyll (−25.37%), which declined to −35.35% after 60 days of refrigerated storage. Interestingly, as reported by Guillén et al. (2016) [54], sous-vide cooking at temperatures below 100 °C is more preservative toward chlorophyll than classic boiling, with less degradation of chlorophylls and a lower concentration of pheophytins generated. Chlorophyll b was more severely affected, decreasing by 54%, while chlorophyll a showed a 25.92% reduction. Although it is generally reported that chlorophyll a is more susceptible to thermal degradation than chlorophyll b, especially under acidic conditions [55,56], Paciulli et al. (2017) [57] exceptions may occur depending on the cooking method or the vegetable matrix. In fact, this pattern of greater degradation of chlorophyll b compared to chlorophyll a is consistent with previous studies. Martínez–Hernández et al. (2013) [2] reported similar results in sous-vide-treated kailan-hybrid broccoli, showing a more pronounced reduction in chlorophyll b after treatment and during cold storage. Gonnella et al. (2018) [50] observed the same trend in asparagus cooked with various methods, noting that even sous-vide led to higher chlorophyll b degradation. Danowska–Oziewicz et al. (2019) have reported similar results in broccoli and asparagus cooked using various methods [58], while Paciulli et al. (2015) [59] observed a 20% degradation of chlorophyll a and a 44% degradation of chlorophyll b in boiled asparagus, Dos Reis et al. (2015) [60] found a 16% and 33% reduction in chlorophyll a and b, respectively, in steamed broccoli, and Korus (2013) reported a greater loss of chlorophyll b than chlorophyll a in canned kale [53].

### 2.3. Total Polyphenol Content and Antioxidant Activity

The total polyphenol content and antioxidant activity of Bimi^®^ broccoli were evaluated in raw samples, immediately after sous-vide processing (t_1_), and following 60 days of refrigerated storage (t_60_). As shown in Table 5, the sous-vide treatment significantly enhanced TPC and all antioxidant activity compared to the raw sample.

Pearson’s correlation analysis revealed a strong positive correlation between TPC and all antioxidant assays tested (FRAP: *r* = 0.9797; ABTS: *r* = 0.9433; DPPH: *r* = 0.9638; *p* < 0.0001). These results underscore the strong contribution of phenolic compounds to the overall antioxidant capacity of Bimi^®^ broccoli. Similar findings were also reported in previous studies on Brassica vegetables after thermal treatment [38,61]. This improvement, which is likely attributed to heat-induced cell wall disruption and membrane breakdown during cooking, enhances the release and extractability of phenolic compounds. A key factor in this process is the thermal inactivation of polyphenol oxidase which effectively prevents the degradation of these compounds, while the vacuum conditions reduce oxidative losses, ensuring food safety and nutritional value. The release of glycosylated phenolics and other bound forms may also contribute to the observed increase in total polyphenol content and antioxidant activity [2,62,63,64,65]. Although a reduction in all antioxidant parameters was observed after 60 days of refrigerated storage (t_60_), the values remained notably higher than those in untreated broccoli. These results are consistent with findings by Kosewska et al. (2018) [65], who reported that sous-vide processing, compared to conventional boiling, was more effective in preserving and increasing antioxidant potential across different vegetables. The sous-vide method limits the leaching of water-soluble nutrients and reduces oxidative degradation. Similarly, Doniec et al. (2022) [66] demonstrated that hydrothermal processing, particularly steaming and sous-vide, enhanced total polyphenol concentrations and antioxidant activity in Brussels sprouts. Martínez–Hernández et al. (2012) [2] also observed an increase in TPC in kailan-hybrid broccoli following sous-vide cooking, while boiling caused a significant reduction of up to 40% compared to raw samples. They reported even higher FRAP than DPPH values, and a progressive decline in antioxidant activity over 45 days of refrigerated storage, findings that align with the present results.

### 2.4. Microbiological and Physicochemical Stability

During the 90-day storage period, pasteurized Bimi^®^ samples maintained microbiological stability under both standard refrigeration (4 °C) and abusive temperature conditions (6 °C for 25 days followed by 10 °C until the end of the tests). *Salmonella* spp. and *Listeria monocytogenes* were not detectable in 25 g of product at all timepoints [67]. β-glucuronidase-positive *Escherichia coli*, coagulase-positive Staphylococci (*S. aureus* and related species), sulfite-reducing *Clostridia*, and yeasts remained consistently below detection limits (<10 CFU/g), regardless of storage conditions or time. Total aerobic mesophilic counts (TAMCs) were undetectable (<10 CFU/g) through day 40. From day 65 onward, a gradual increase was observed under both conditions: values remained below 10^3^ CFU/g at first and then reached 1.5 × 10^3^ CFU/g (standard) and 3.0 × 10^3^ CFU/g (accelerated) by day 80, peaking at day 90 with 2.2 × 10^3^ CFU/g and 4.6 × 10^3^ CFU/g, respectively. From a physicochemical standpoint, pH decreased progressively from 6.02 at baseline to 5.92 (standard) and 5.80 (abusive temperature condition), while titratable acidity increased from 0.38% to 0.64% and 0.72%, respectively. Aw remained high and stable across all samples (0.986–0.990).

### 2.5. Sensory Evaluation

**Figure 4:**
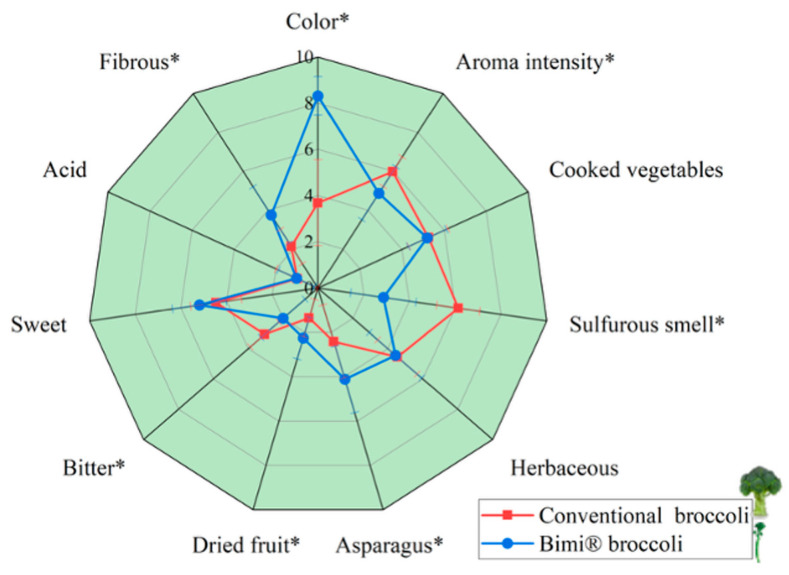
Spider plot representation of the sensory profiles of steamed conventional broccoli and Bimi^®^ broccoli. Attributes marked with an asterisk (*) indicate significant differences between samples (independent-sample *t*-test, *p* ≤ 0.05).

The sensory characteristics of conventional broccoli and Bimi^®^ broccoli were evaluated and are presented in Figure 4. Statistically significant differences were observed between the two types for several descriptors (*p* ≤ 0.05). Bimi^®^ broccoli was perceived as having a more intense color (8.31 ± 0.83 vs. 3.68 ± 1.86), greater fibrousness (3.75 ± 1.45 vs. 2.13 ± 0.88), and a stronger asparagus-like note (4.11 ± 1.50 vs. 2.41 ± 1.64) compared to conventional broccoli. The higher fibrousness in Bimi^®^ can be attributed to the fact that it is consumed whole, including the stem. In contrast, the stem of conventional broccoli is typically discarded due to its toughness. Indeed, the edible portion of common broccoli accounts for only about 51% of the whole (CREA, 2019) [68], while for Bimi^®^, it reaches 100% [12]. The greater fibrousness recorded in Bimi^®^ is therefore linked to the presence of the stem, which, however, is known to be softer and more similar to asparagus, making it completely edible compared to the woody stem of traditional broccoli [69,70]. In contrast, conventional broccoli scored significantly higher in aroma intensity (5.98 ± 0.76 vs. 4.87 ± 1.36), sulfurous odor (6.13 ± 0.94 vs. 2.86 ± 1.43), herbaceous notes (4.55 ± 1.13 vs. 4.44 ± 1.50), and bitterness (3.06 ± 0.82 vs. 2.00 ± 1.26). This is particularly relevant given that many consumers find conventional broccoli to have a bitter taste and sulfurous notes [10]. Although Bimi^®^ also showed a slightly higher sweetness score (5.19 ± 1.18 vs. 4.46 ± 1.11), the differences in cooked vegetable aroma (5.25 ± 0.93 vs. 5.20 ± 0.90), sweetness, acidity (0.98 ± 1.01 vs. 1.02 ± 0.86), and dried fruit notes (1.35 ± 0.84 vs. 2.25 ± 0.94) were not statistically significant.

## 3. Materials and Methods

### 3.1. Chemicals and Reagents

All chemicals and solvents used are of analytical grade. Ultrapure water (resistivity 18.2 M Ω cm at 25 °C) was obtained from a Milli–Q system (Millipore Corporation, Milan, Italy). Reagents including sodium hydroxide, hydrochloric acid, ethanol, methanol, iron (III) chloride, TPTZ, sodium carbonate, Folin–Ciocalteu reagent, gallic acid, Trolox, sodium acetate, acetic acid, DPPH, acetone, sulfuric acid, nitric acid, barium hydroxide, and sodium methoxide were sourced from Sigma–Aldrich (St. Louis, MO, USA). The ABTS Assay Kit (KF01002) was purchased from Bioquochem (Oviedo, Asturias, Spain). Derivatization reagents (AccQTag Ultra Kit and AQC) were obtained from Waters (Milford, CT, USA). All microbiological culture media were supplied by Diagnostic International Distribution (Milan, Italy).

### 3.2. Sample Preparation and Industrial Processing

Bimi^®^ (kailan hybrid, *Brassica oleracea* Italica × *Alboglabra* group) was supplied by its Italian licensee, Cinana Vibes (Cesena, Italy). After collection, samples were chilled with crushed ice and transported under refrigerated conditions (4–8 °C) to Ghisetti 1870 Srl (Badia Polesine, Rovigo, Italy), a fifth-range vegetable processing company. Upon arrival, the samples was stored at 4 °C and processed within 24 h. For sensory comparison, conventional broccoli (*Brassica oleracea*, Italica group) was purchased from a local market (Lusia, Rovigo, Italy), processed within the same day, and stored under the same conditions. Samples underwent a standardized pre-treatment process consisting of visual inspection, removal of non-compliant parts, and a two-step washing procedure: an initial rinse in potable water followed by immersion in chlorinated water (100 mg L^−1^ free chlorine, pH 6.5, 5 °C), and a final rinse with clean water. After drying to reduce surface moisture, Bimi^®^ samples were processed in their entirety—including florets and stems—as the stem is tender and fully edible. In contrast, the basal woody portion of the conventional broccoli stem was removed prior to processing, in accordance with standard culinary practices. All samples were portioned into uniform 150 ± 5 g units and vacuum-sealed at 20 mBar using a thermoforming system (EVO 7000, Tecnosistem S.r.l., Brescia, Italy) with PE/PA multilayer film (Amilen KF, Niederwieser S.p.a., Modena, Italy Sealed Air S.r.l., Milan, Italy). Packaged units were pasteurized in an industrial autoclave (ST1200 × 4000 1D S–WR–SA, Levati Food Tech, Parma, Italy) by applying a thermal treatment equivalent to P_90_ ≥ 10 min at the product’s geometric center, ensuring 6–log reduction in non-proteolytic *Clostridium botulinum* spores [24,25,26,27]. The core temperature of the product was monitored throughout pasteurization using an S–micro L 20 data logger (Tecnosoft S.r.l., Milan, Italy), and data were recorded every 10 s and managed using SPD software (version 1.7.2.0). Rapid cooling followed immediately to reduce the internal temperature to 4 °C. Fresh unprocessed Bimi^®^ samples, along with sous-vide pasteurized Bimi^®^, were transported to the university laboratories under refrigerated conditions (4–8 °C) and subsequently analyzed. Chemical analyses were conducted on both raw and sous-vide pasteurized Bimi^®^ samples. For the raw, unprocessed product, a comprehensive nutritional profile was determined. Both raw and processed samples were also evaluated for total phenolic content, antioxidant activity, chlorophyll content, and colorimetric values. Analyses on thermally treated samples were carried out both the day after processing and after 60 days of refrigerated storage (4 °C), which corresponds to the commercial shelf life currently defined by the company. Microbiological analyses were extended to 0, 40, 65, 80, and 90 days to monitor microbial stability over time. Sensory profiling and consumer acceptance tests were carried out one day post-processing, comparing sous-vide-treated Bimi^®^ broccoli and conventional broccoli processed under identical thermal conditions.

### 3.3. Compositional Characterization of Raw Bimi^®^ Broccoli

#### 3.3.1. Proximate Composition

Composition analysis of Bimi^®^ was conducted in triplicate according to the AOAC procedures [71]. Moisture content (AOAC 934.01) was determined gravimetrically by drying to a constant weight at 105 °C. The ash content (AOAC 942.05) was determined by incineration of approximately 5 g of sample in a porcelain pan in a muffle at 550 °C for 6 h. Crude protein was quantified using the Kjeldahl method with a nitrogen-to-protein conversion factor of 6.25 (AOAC 2001.11). Crude fiber (AOAC 2001.11) was assessed by sequential acid and alkali digestion: Samples were boiled in 0.26 M sulfuric acid for 30 min, followed by filtration, rinsing, and subsequent boiling in 0.31 M sodium hydroxide. After a second filtration and rinse, the residue was dried at 130 °C for 2 h and incinerated at 350 °C to determine weight loss. Crude fat content (AOAC 991.36) was measured using the Soxhlet extraction method with a Soxtec™ 2046 system (FOSS, Hillerød, Denmark). The availability of carbohydrates, different from fiber, was estimated by difference [72,73] using the following formula (Equation (1)):

Carbohydrates (%) = 100 − [moisture (%) + protein (%) + fat (%) + ash (%) + fiber (%)](1)

The Bimi ^®^ energy evaluation, in kcal/100 g, was performed using conversion factors based on general Atwater factors for food energy content in EU Regulation N. 1169, 2011 [30], these being 4.0 kcal/g for protein and carbohydrates, 9.0 kcal/g for fat, and 2.0 kcal/g for fiber. Data are expressed as grams per 100 g of fresh weight (g/100 g FW).

#### 3.3.2. Mineral Content

The concentration of selected macroelements (Ca, K, Mg, Na, P, and S) in Bimi^®^ broccoli was determined as described by Lante et al. (2006) [74]. Analyses were carried out using an inductively coupled plasma optical emission spectrometer (ICP–OES, SPECTRO CIROS^CCD^, Spectro Analytical Instruments, Kleve, Germany) equipped with axial plasma viewing. Samples (~0.5 g) were mineralized in a closed-vessel microwave digestion system (Ethos 1600 Milestone S.r.l., Sorisole, Bergamo, Italy) using a mixture of 5.0 mL of ultrapure nitric acid (HNO_3_, 70%) and 1.5 mL of hydrogen peroxide (H_2_O_2_, 30% *v*/*v*). After digestion, the solutions were cooled, filtered through 0.45 µm membrane filters, and brought to a final volume of 25.0 mL with ultrapure water. Calibration curves were prepared using certified single-element standard solutions (Spectrascan, Teknolab A/S, Ski, Norway). The ICP–OES was operated under the following conditions: RF power 1400 W, plasma gas flow rate 13.5 L/min, auxiliary gas 1.2 L/min, nebulizer gas 0.90 L/min, sample aspiration rate 2.0 mL/min, preflush time 45 s, and read time 3 × 24 s. Data are expressed as milligrams per 100 g of fresh weight (mg/100 g FW).

#### 3.3.3. Amino Acid Profile Determination

The amino acid profile was determined following a protocol adapted from the European Pharmacopoeia, as described by Ebrahimi et al. (2022) [75], with slight modifications. Analyses were conducted using a high-performance liquid chromatography (HPLC) system (Agilent 1260 Infinity, Agilent Technologies, Santa Clara, CA, USA) equipped with a diode array detector (DAD VL+) and a CORTECS C18 reversed-phase column (2.7 µm, 2.1 × 150 mm), maintained at 45 °C. The procedure included acid hydrolysis and pre-column derivatization with 6–aminoquinolyl–N–hydroxysuccinimidyl carbamate (AQC), as reported by Bosch et al. (2006) [76]. For the analysis of amino acids, proteins were hydrolyzed in 6 M hydrochloric acid (HCl) at 105 °C for 24 h. To quantify cysteine (Cys), samples underwent pre-hydrolysis oxidation with 3,3′–dithiobispropionic acid to form a stable disulfide derivative. Post-hydrolysis, samples were neutralized with 8 M sodium hydroxide (NaOH), adjusted to the appropriate pH, and filtered through 0.45 µm filters. Derivatization was carried out by mixing 70 µL of AccQTag Ultra borate buffer with 10 µL of the filtered hydrolysate, followed by the addition of 20 µL of AQC reagent (dissolved in acetonitrile). The mixture was vortexed and incubated at 55 °C for 10 min, and then diluted with 900 µL of borate buffer. A 5 µL aliquot was injected into the HPLC system for analysis. Tryptophan (Trp) was determined using a method adapted from Directive 2000/45/EC [77]. Briefly, 100 mg of sample was hydrolyzed in Teflon tubes with 8 g of barium hydroxide and 16 mL of distilled water at 105 °C for 24 h. After cooling, the hydrolysate was neutralized to pH 7 using 6 N HCl and diluted to 100 mL with 1 M sodium borate buffer. Samples were filtered through 0.22 µm syringe filters, and 20 µL was injected into an XSelect HSS T3 column (5 µm, 4.6 × 250 mm). Chromatographic separation was carried out under isocratic conditions using a mobile phase of 25 mM sodium acetate and acetonitrile (91:9, *v*/*v*), at a flow rate of 0.9 mL/min. Detection was performed at 280 nm using a DAD. Data are expressed as milligrams per 100 g of fresh weight (mg/100 g FW).

#### 3.3.4. Fatty Acid Profile Determination

The fatty acid composition was determined by comprehensive two-dimensional gas chromatography (GC × GC) using an Agilent 7890A gas chromatograph (Agilent Technologies, Santa Clara, CA, USA) equipped with an Agilent 7683 autosampler, a flame ionization detector (FID), and a capillary flow technology (CFT) modulator. Sample preparation involved the transesterification of lipids into fatty acid methyl esters (FAMEs). Approximately 40 mg of sample was treated with 1 mL of 0.5 M sodium methoxide in methanol and incubated at 50 °C for 15 min. After cooling to room temperature, 1.5 mL of methanolic HCl (5%) was added, and the mixture was incubated at 80 °C for an additional 15 min. Once cooled again, 2 mL of hexane and 2 mL of 6% potassium carbonate solution were added. The mixture was vortexed for 30 s and centrifuged at 4000× *g* for 5 min at 4 °C. The resulting upper organic phase containing FAMEs was recovered and subjected to chromatographic analysis. One microliter of the supernatant was injected into the GC system in split mode (split ratio 160:1), with hydrogen as the carrier gas. Chromatographic separation was performed using a Supelco SP–2560 column (Merck, Darmstadt, Germany) as the first dimension and an Agilent J&W HP–5 ms column as the second. The oven temperature was initially set at 40 °C for 2 min, increased to 170 °C at 50 °C/min (held for 25 min), then ramped to 250° C at 2 °C/min, and held for 14 min. Injector and detector temperatures were maintained at 270 °C and 300 °C, respectively. Data acquisition and processing were carried out using GC × GC Image R 2.2 software (Zoex Corp., Houston, TX, USA). Fatty acids were identified using high-purity reference standards (Sigma–Aldrich, Merck, Milano, Italy) and are expressed as relative percentages of the total fatty acid content.

### 3.4. Color Assessment

Colorimetric analyses were performed on both florets and stems of Bimi^®^ broccoli to evaluate color attributes throughout processing and storage. Prior to analysis, samples were removed from packaging, allowed to equilibrate at room temperature (~20 °C) for 20 min, and gently blotted to remove surface moisture. Color was measured using the Chroma Meter CR–300 colorimeter (Konica Minolta, Milan, Italy) with a port (Ø 8 mm) and the D65 illuminant, and standard observer 2°. The chromameter was calibrated using a standardized white tile before measurement. For each sampling point (raw, post-processing, and after 60 days of storage), three individual Bimi^®^ units (*n* = 3) were randomly selected. On each unit, five spot measurements were taken at equidistant positions on both florets and stems [7], and the mean value of each unit was then used as a replicate. The color parameters are expressed as L* (100 lightness/darkness 0), a* (+*a* redness/greenness −*a*), and b* (+*b* yellowness/blueness −*b*) in the CIE Lab system (The International Commission on Illumination). Hue angle (*H*° = arctg b*/a*) and chroma (*C* = [a*^2^ + b*^2^]^1/2^) were then calculated from primary L*, a*, and b* readings. The total color difference (Δ*E*) was also calculated to assess the impact of sous-vide treatment and storage on broccoli color, as described by Gonnella et al. (2018) [50]. 

### 3.5. Chlorophyll Determination

The total chlorophyll and the chlorophyll a and b contents were determined according to the AOAC method (942.04) [78]. The sample (5 g) was homogenized with 25 mL of acetone/water (85:15 *v*/*v*) and 0.1 g of CaCO_3_ and then filtered and re-extracted until the tissue was devoid of any green and the washing solvent remained colorless. The extract was centrifuged (5000 rpm, 10 min). Appropriate dilution of supernatant was used to read the absorbance with an Agilent Cary 60 UV–Vis spectrophotometer (Agilent Technologies, Santa Clara, CA, USA) at 660.0 nm and 642.5 nm. From the absorbance values, chlorophyll (*a*, *b*, and total) was calculated as reported by Chiu and Lai (2010) [79] and is expressed as mg/100 g fresh weight.

### 3.6. Extraction of Samples for Total Phenolic Content and Antioxidant Activity

Ethanolic extraction for TPC, FRAP, ABTS, and DPPH assays was carried out based on the method of Ismail et al. (2004) [80] with slight modifications. Briefly, 100 g of sample was chopped and homogenized (Silvercrest^®^ SSMS 600 E6, Bochum, Germany) for 2 min, and then mixed with 70% ethanol at a 1:5 ratio. The mixture was stirred at 200 rpm on an orbital shaker for 1 h at room temperature and filtered through Whatman No. 1 paper. The residue was re-extracted twice under the same conditions, and all filtrates were combined.

### 3.7. Determination of Total Phenolic Content

TPC was quantified using the Folin–Ciocalteu method as described by Cisneros–Yupanqui et al. (2021) [81]. Briefly, 1 mL of ethanolic extract was mixed with 5 mL of NaCO_3_ 10%, containing NaOH 1 M, and 500 µL of Folin–Ciocalteau’s reagent previously diluted twice in distilled water. A blank solution was also prepared with the dilution solvent. After waiting 30 min under darkness, the samples were filtered using 0.45 µm acetate cellulose filters (Millipore, Bedford, MA, USA) and the absorbance value was measured at 650 nm with an Agilent Cary 60 UV–Vis spectrophotometer (Agilent Technologies, Santa Clara, CA, USA). TPC was calculated from the standard curve of gallic acid (R^2^ = 0.998), and the results are expressed as mg gallic acid equivalents (GAE) per 100 g FW.

### 3.8. Antioxidant Activity

The antioxidant activity was assessed using FRAP (Ferric Reducing Antioxidant Power), DPPH (2,2–diphenyl–1–picrylhydrazyl), and ABTS (3–ethyl–benzothiazoline–6–sulfonic acid) assays. The FRAP assay was carried out according to Benzie and Strain (1996) [82], by mixing 300 mM acetate buffer (pH 3.6), 10 mM TPTZ in 40 mM HCl, and 20 mM FeCl_3_ in a 10:1:1 ratio. A volume of 0.9 mL of the FRAP reagent was combined with 0.1 mL of extract and incubated at 37 °C for 30 min, and absorbance was recorded at 593 nm. The DPPH assay, following Ebrahimi et al. (2024) [83], involved mixing 0.1 mL of extract with 0.9 mL of 0.1 mM DPPH solution in ethanol. After incubation at 22 °C for 30 min in the dark, absorbance was measured at 517 nm. The ABTS assay was conducted using a commercial assay kit (Ref. Kf-01-002, Bioquochem SL, Llanera, Spain), and absorbance was read at 734 nm, according to the manufacturer’s protocol. All absorbance measurements were performed using an Agilent Cary 60 UV–Vis spectrophotometer (Agilent Technologies, Santa Clara, CA, USA). Results were calculated using Trolox standard curves (R^2^ ≥ 0.99) and are expressed as mmol Trolox equivalents (TE) per 100 g fresh weight (FW).

### 3.9. Microbial and Physicochemical Analyses

Analyses were conducted on pasteurized Bimi^®^ samples at 0, 40, 65, 80, and 90 days under two storage conditions: standard storage (4 °C) and in abusive temperature conditions (6 °C for 25 days, then 10 °C until the end of the test). The physicochemical parameters measured included pH value (Crison GLP 22 pH–meter, Barcelona, Spain), activity water (a_w_) values (Novasina^®^ LabMaster, Lachen, Switzerland), and titratable acidity determined as citric acid in grams per 100 g of sample. Microbiological analyses covered total mesophilic count (UNI EN ISO 4833–1:2013/Amd 1:2022) [84], coliforms (ISO 4832:2006) [85], *Escherichia coli* β-glucuronidase-positive (ISO 16649–2:2001) [86], coagulase-positive Staphylococci (UNI EN ISO 6888-2:2021/Amd 1:2023) [87], sulfite–reducing *Clostridia* (ISO 15213–1:2023) [88], mesophilic lactic acid bacteria (ISO 15214:1998) [89], and yeasts (ISO 21527–1:2008) [90]. *Salmonella* spp. and *Listeria monocytogenes* were detected by real-time PCR using iQ-Check™ kits (Bio-Rad, Hercules, CA, USA), validated by AFNOR (certificate numbers BRD 07/06–07/04 and BRD 07/10–04/05, respectively). Colonies were counted and results are reported as colony forming units per g (CFU/g) of sample. 

### 3.10. Sensory Profile of Bimi^®^ Compared to Conventional Broccoli

To evaluate the sensory characteristics of Bimi^®^ following industrial sous-vide processing, a Quantitative Descriptive Analysis (QDA^®^) was performed. The objective was to characterize the sensory profile of Bimi^®^ and compare it to conventional broccoli, which served as a reference product and was processed under identical thermal conditions. Twelve trained panelists (6 women and 6 men, aged 26–56), all experienced in sensory evaluation of vegetables, carried out the analysis in a dedicated sensory laboratory under controlled lighting and temperature conditions. The panelists underwent a 1-month training period, in which they acquired familiarity with the product, and generated and agreed on attributes. Once the panel became sufficiently familiar with the specific vocabulary, and the evaluation protocol was established, the panel conducted a second phase of training, focusing on quantitative evaluation to acquire familiarity with the use of the intensity scale (range 0–10). For the study, samples of Bimi^®^ and conventional broccoli (20 g portions) were slightly reheated prior to evaluation and served at room temperature. Panelists assessed eleven attributes: green color intensity, aroma intensity, cooked vegetable, sulfurous, herbaceous, asparagus, dried fruit, bitterness, sweetness, acidity, and fibrousness. Sensory ratings were collected using a 15 cm unstructured line scale, anchored at 0 (“low intensity”) and 10 (“high intensity”) for each descriptor. Between samples, panelists cleansed their palates with water and unsalted crackers.

### 3.11. Data Analysis

Data are expressed as mean values ± standard deviation (SD). Data collection was performed using Microsoft Excel 365 (Microsoft Corporation, Redmond, DC, USA), while sensory evaluation data were acquired using FIZZ Biosystems software (version 2.51 c 02, Biosystèmes, Couternon, Bourgogne, France). Statistical analyses were conducted using Microsoft Excel and OriginPro^®^ 2024 (OriginLab Corporation, Northampton, MA, USA). Statistical differences were evaluated using one-way ANOVA with Tukey’s post hoc test for comparisons among three or more groups, and independent two-sample *t*-tests (with Welch correction if variances were unequal) for two-group comparisons. Pearson correlation coefficients (*r*) were calculated to assess the relationship between TPC and antioxidant activity (FRAP, ABTS, DPPH). All tests were performed at a 95% confidence level (*p* ≤ 0.05). All graphical representations were generated using OriginPro^®^ 2024.

## 4. Conclusions

This study provides comprehensive insights into the compositional, nutritional, and sensory characteristics of Bimi^®^ broccoli, both fresh and after industrial sous-vide processing. Microbiological stability was confirmed over 90 days of refrigerated storage under both standard and abusive temperature conditions. *Listeria monocytogenes* and *Salmonella* spp. were consistently undetectable in 25 g of product across all timepoints. Spoilage and hygiene indicators such as *Enterobacteriaceae* and lactic acid bacteria remained below the detection limit (<10 CFU/g) throughout the storage period. Total aerobic mesophilic counts stayed negligible until day 40, and by day 90 reached 2.7 × 10^3^ CFU/g under standard conditions and 4.6 × 10^3^ CFU/g under abusive conditions—both within acceptable safety thresholds. Physicochemical parameters also showed good stability. Water activity remained high and constant (0.986–0.990) under all conditions. pH decreased slightly from 6.02 to 5.92 (standard) and 5.80 (abusive), while titratable acidity increased from 0.38% to 0.64% and 0.72%, respectively, indicating mild acidification over time. Thermal processing improved the release and extractability of phenolic compounds and antioxidant constituents. Following sous-vide treatment, total polyphenol content rose from 22.72 ± 0.39 to 39.44 ± 0.71 mg GAE/100 g FW and remained elevated (27.64 ± 0.98 mg GAE/100 g FW) after 60 days of refrigerated storage. Antioxidant capacity followed a comparable trend: FRAP values more than doubled post-treatment (from 1.76 ± 0.09 to 4.03 ± 0.14 mmol TE/100 g FW), while ABTS and DPPH assays similarly indicated marked increases. Although a progressive decline in antioxidant values was observed over time, all indices remained significantly higher than in raw samples at day 60. In contrast, the sous-vide processing caused a significant loss of total chlorophyll (−25.37%), which declined to −35.35% after 60 days of refrigerated storage.

From a sensory perspective, Bimi^®^ demonstrated distinct advantages over conventional broccoli. It was rated significantly lower in bitterness and sulfurous notes, and exhibited a more delicate flavor profile, marked by asparagus-like aromatic notes and a fibrous texture associated with the full edibility of the stem. These features may enhance consumer acceptance, particularly among those seeking milder-tasting Brassica options.

Altogether, these findings position Bimi^®^ as a promising health-oriented convenience food that is well aligned with the demands of modern consumers, instilling confidence in its market acceptance.

Future research should explore the stability and bioavailability of bioactive compounds in Bimi^®^ during processing and storage, focusing on their functional relevance and impact on overall product quality. In particular, glucosinolates and their enzymatic or thermal degradation products represent a key class of compounds that merit further investigation, due to their well-established health-promoting properties in Brassica vegetables. From a product development perspective, integrating Bimi^®^ into diverse convenience food formats presents promising opportunities for innovation and product valorization. More importantly, Bimi^®^ aligns with current consumer trends, providing strong market potential and reassurance for its future success.

## Figures and Tables

**Table 1:** Proximate composition (g/100 g FW) of Bimi^®^ broccoli.

Parameter	(g/100 g FW)
Moisture	89.39 ± 0.56
Ash	0.84 ± 0.01
Fats	0.46 ± 0.04 (0.66% RDA)
Protein	4.32 ± 0.06 (8.63% RDA)
Carbohydrates	2.03 ± 0.12 (0.78% RDA)
Dietary fiber	2.96 ± 0.03 (11.85% RDA)
Energy (kcal/100 g)	35.46 ± 0.14 (1.77% RDA)

Values are presented as mean ± SD, *n* = 3. The proximate composition of Bimi^®^ broccoli is correlated with RDA values.

**Table 2:** Minerals (mg/100 g FW) in different fractions of raw Bimi^®^ broccoli.

Minerals	Whole	Floret	Stem
Ca	64.75 ± 5.68 ^b^	87.74 ± 4.61 ^a^	51.77 ± 1.57 ^c^
K	311.79 ± 11.54 ^a^	249.23 ± 24.53 ^b^	357.66 ± 19.75 ^a^
Mg	18.55 ± 0.54 ^b^	22.36 ± 0.87 ^a^	15.53 ± 0.95 ^c^
Na	3.87 ± 0.75 ^b^	3.49 ± 0.29 ^b^	4.96 ± 0.14 ^a^
P	98.32 ± 4.13 ^a^	106.90 ± 6.38 ^a^	84.94 ± 2.52 ^b^
S	143.38 ± 6.17 ^ab^	164.21 ± 17.72 ^a^	122.36 ± 6.76 ^b^

Values are presented as mean ± SD, *n* = 3. Different superscript letters within the same row indicate significant differences among the fractions (*p* ≤ 0.05), according to one-way ANOVA followed by Tukey’s HSD post hoc test.

**Table 3:** Amino acid composition (mg/100 g FW) of different fractions of Bimi^®^ broccoli.

Amino Acid	Whole	Floret	Stem
Essential amino acids			
Histidine	75.99 ± 0.74 ^b^	114.29 ± 3.69 ^a^	56.53 ± 3.62 ^c^
Isoleucine	81.42 ± 2.51 ^b^	119.77 ± 3.64 ^a^	64.26 ± 0.56 ^c^
Leucine	134.93 ± 2.13 ^b^	203.50 ± 1.48 ^a^	98.53 ± 7.00 ^c^
Lysine	183 ± 4.67 ^b^	276.87 ± 20.23 ^a^	126.21 ± 0.59 ^c^
Methionine	17.01 ± 0.65 ^a^	18.40 ± 0.93 ^a^	13.36 ± 0.94 ^b^
Phenylalanine	96.20 ± 2.63 ^b^	143.85 ± 5.67 ^a^	71.56 ± 2.46 ^c^
Threonine	96.48 ± 2.85 ^b^	140.43 ± 12.56 ^a^	75.63 ± 5.57 ^c^
Tryptophan	29.32 ± 0.36 ^b^	52.65 ± 1.87 ^a^	21.38 ± 1.43 ^c^
Valine	129.63 ± 3.86 ^b^	176.46 ± 2.04 ^a^	105.04 ± 8.15 ^c^
Total EAA	844.01 ± 4.16 ^b^	1246.23 ± 40.39 ^a^	632.51 ± 4.99 ^c^
Non-essential amino acids			
Arginine	207.05 ± 10.19 ^b^	357.80 ± 22.18 ^a^	136.08 ± 1.60 ^c^
Alanine	123.11 ± 0.41 ^b^	156.01 ± 14.60 ^a^	101.21 ± 4.46 ^b^
Aspartic acid	489.76 ± 46.10 ^a^	520.39 ± 36.53 ^a^	405.78 ± 56.79 ^a^
Cysteine	27.18 ± 0.92 ^b^	38.76 ± 2.09 ^a^	22.93 ± 0.47 ^c^
Glutamic acid	1761.96 ±88.33 ^a^	1618.68 ± 130.08 ^a^	1743.10 ± 123.47 ^a^
Glycine	95.72 ± 3.60 ^b^	147.42 ± 5.58 ^a^	77.81 ± 2.51 ^c^
Proline	98.81 ± 3.93 ^b^	127.19 ± 15.75 ^a^	83.65 ± 6.18 ^b^
Serine	138.55 ± 3.50 ^b^	183.87 ± 22.97 ^a^	111.45 ± 4.27 ^b^
Tyrosine	49.45 ± 2.38 ^b^	74.60 ± 6.84 ^a^	39.31 ± 3.07 ^b^
Total NEAA	2991.59 ± 46.24 ^ab^	3224.70 ^a^	3224.70 ± 156.26 ^b^
Total amino acids	3835.60 ± 49.52 ^b^	4470.92 ± 196.52 ^a^	3353.83 ±193.11 ^c^

Values are presented as mean ± SD, *n* = 3. Different superscript letters within the same row denote significant differences among the fractions (*p* ≤ 0.05, one–way ANOVA + Tukey’s test).

**Table 4:** Color parameters (L*, a*, b*, C*, *H°*, Δ*E*) of Bimi^®^ broccoli stems and florets in raw, sous-vide t_1_, and sous-vide t_60_ samples.

Sample		L*	a*	b*	C*	*H°*	∆*E*
Stem							
Raw sample	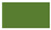	48.56 ± 0.38 ^a^	– 20.06 ± 0.38 ^a^	33.27 ± 0.11^b^	38.85 ± 0.15 ^a^	120.68 ± 0.44 ^a^	–
Sous-vide t_1_	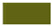	40.30 ± 0.23 ^c^	–10.43 ± 0.50 ^c^	35.52 ± 0.46 ^a^	37.02 ± 0.50 ^b^	106.89 ± 0.43 ^b^	12.89 ± 0.35 ^c^
Sous-vide t_60_	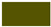	35.45 ± 0.24 ^e^	– 8.75 ± 0.73 ^d^	36.03 ± 0.62 ^a^	37.09 ± 0.44 ^b^	103.80 ± 1.30 ^c^	17.54 ± 0.38 ^a^
Floret							
Raw sample	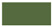	42.75 ± 0.38 ^b^	–12.65 ± 0.51^b^	20.28 ± 0.32 ^d^	23.91± 0.31 ^e^	121.71 ± 1.29 ^a^	–
Sous-vide t_1_	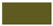	37.35 ± 0.28 ^d^	–6.38 ± 0.18 ^e^	28.42 ± 0.26 ^c^	29.13 ± 0.26 ^d^	102.33 ± 0.66 ^c^	11.51 ± 0.26 ^d^
Sous-vide t_60_	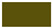	34.36 ± 0.14 ^f^	–5.33 ± 0.45 ^e^	32.77 ± 0.21 ^b^	33.20 ± 0.14 ^c^	98.73 ± 1.71 ^d^	16.73 ± 0.32 ^b^

Values are mean ± SD (*n* = 3). t_1_ and t_60_ indicate 1 and 60 days of refrigerated storage at 4 °C post sous-vide processing. Different superscript letters denote significant differences (*p* ≤ 0.05, one-way ANOVA + Tukey’s test).

**Table 5:** TPC, FRAP, ABTS, and DPPH of Bimi^®^ broccoli samples: raw, after 1 day (t_1_), and after 60 days (t_60_) of refrigerated storage at 4 °C post sous-vide processing.

	TPC	FRAP	ABTS	DPPH
	(mg GAE/100 g FW)	(mmol TE/100 g FW)		
Raw sample	22.72 ± 0.39 ^c^	1.76 ± 0.09 ^c^	1.02 ± 0.16 ^c^	0.40 ± 0.07 ^c^
Sous-vide t_1_	39.44 ± 0.71 ^a^	4.03 ± 0.14 ^a^	3.25 ± 0.10 ^a^	1.64 ± 0.05 ^a^
Sous-vide t_60_	27.64 ± 0.98 ^b^	2.69 ± 0.11 ^b^	2.27 ± 0.16 ^b^	1.02 ± 0.05 ^b^

Values are mean ± SD (*n* = 3). Results are expressed as mg gallic acid equivalents (GAE) per 100 g FW and as mmol Trolox equivalents (TE) per 100 g fresh weight (FW). Different superscript letters within the same column indicate significant differences between treatments (*p* ≤ 0.05, ANOVA followed by Tukey’s test).

## Data Availability

The authors declare that the data supporting the findings of this study are available within the paper. Should any raw data files be needed in another format, they will be available from the corresponding author upon reasonable request. The data are not publicly available due to privacy.

## Abbreviations

The following abbreviations are used in this manuscript:

ABTS	3–ethylbenzothiazoline–6–sulfonic acid
AOAC	Association of Official Analytical Collaboration
AQC	6–aminoquinolyl–N–hydroxysuccinimidyl carbamate
Aw	Activity Water
CFU	Colony-Forming Unit
CFT	Capillary Flow Technology
DAD	Diode Array Detector
DPPH	2,2–diphenyl–1–picrylhydrazyl
EAAs	Essential Amino Acids
FAMEs	Fatty Acid Methyl Esters
FID	Flame Ionization Detector
FRAP	Ferric Reducing Antioxidant Power
FW	Fresh Weight
GAE	Gallic Acid Equivalents
GC×GC	Comprehensive Two–Dimensional Gas Chromatography
HPLC	High-Performance Liquid Chromatography
ICP–OES	Inductively Coupled Plasma Optical Emission Spectrometry
MUFAs	Monounsaturated Fatty Acids
NEAAs	Non-Essential Amino Acids
PUFAs	Polyunsaturated Fatty Acids
QDA^®^	Quantitative Descriptive Analysis
RDA	Recommended Dietary Allowance
SFAs	Saturated Fatty Acids
SD	Standard Deviation
TE	Trolox Equivalents
TPC	Total Phenolic Content
TPTZ	2,4,6–tri(2–pyridyl)–s–triazine
